# Characteristics and pregnancy outcomes of patients with severe pneumonia complicating pregnancy: a retrospective study of 12 cases and a literature review

**DOI:** 10.1186/s12884-018-2070-0

**Published:** 2018-11-03

**Authors:** Pingping Tang, Jiangshan Wang, Yingna Song

**Affiliations:** 1 Department of Obstetrics and Gynecology, Peking Union Medical College Hospital, Chinese Academy of Medical Sciences and Peking Union Medical College, Beijing, China; 2 Department of Emergency, Peking Union Medical College Hospital, Chinese Academy of Medical Sciences and Peking Union Medical College, Beijing, China

**Keywords:** Severe pneumonia, Pregnancy, Pregnancy outcomes, Perinatal outcomes

## Abstract

**Background:**

Pneumonia during pregnancy has been proven to be associated with increased maternal and fetal morbidity and mortality. The management of severe pneumonia in gravid patients is even more challenging. Thus, we summarized the characteristics and pregnancy outcomes of these patients and explored the probable risk factors and predictive factors for pneumonia during pregnancy and the appropriate timing of delivery in severe pneumonia patients.

**Methods:**

A retrospective cohort study was conducted with 12 patients who were diagnosed with severe pneumonia complicating pregnancy at Peking Union Medical College Hospital between January 2010 and June 2017. The clinical features, treatment strategies, and pregnancy outcomes were collected from medical records and telephone calls.

**Results:**

All 12 patients were in their late second or third trimester. The patients had a higher prevalence of anemia (50%) and preeclampsia (25%) than ordinary pregnant women. Delayed diagnoses were not uncommon. Two mothers died in our series, resulting in a mortality rate of 17%. Two intrauterine deaths were observed. Elective delivery was not performed in any of the four patients in their second trimester. Six of the seven patients who presented after 28 weeks of gestation and had live fetuses underwent emergency deliveries. Preterm births (6/7) and cesarean sections (5/7) were the two leading adverse outcomes in newborns.

**Conclusions:**

Anemia, advanced gestational age, and preeclampsia might be associated with the severity of pneumonia. Chest radiographs should be taken as soon as pneumonia is highly suspected to facilitate an early diagnosis. High incidences of adverse fetal outcomes were observed; thus, termination of the pregnancy is recommended for patients in their third trimester when respiratory function deteriorates progressively. However, it might be reasonable to continue pregnancy for those in their first or second trimester.

## Background

Pneumonia is the most common cause of fatal nonobstetric infections in pregnant patients [1]. In the United States, pneumonia complicates approximately 1 per 1000 pregnancies [2]. In Taiwan, pneumonia complicates 6.7 per 1000 pregnancies [3]. In mainland China, this rate may be even higher. A true estimate of the incidence of severe pneumonia during pregnancy is difficult to obtain because the few published studies in this area are mostly case reports.

Considering the potential teratogenicity of radiography and drugs, both patients and their physicians tend to postpone radiation examinations and medical treatment when pneumonia is suspected. As a result, delayed diagnoses and referral are common in patients with pneumonia complicating pregnancy [2]. Several physiological and immunological changes that are experienced during pregnancy, such as altered T lymphocyte immunity, increased oxygen consumption, decreased functional residual capacity, decreased chest compliance, and increased risk of aspiration, may predispose pregnant women to a more severe course of pneumonia, which may result in greater maternal and fetal morbidity and mortality [1, 4].

A meta-analysis revealed that influenza infection in pregnant individuals resulted in a higher risk of hospital admission than influenza infection in nonpregnant individuals [5]. Early recognition and prompt treatment of severe pneumonia are essential to improve maternal and perinatal outcomes [6]. Because of the complexity of such cases and the ethical considerations, no randomized controlled trials have been performed in this area, and few high-quality studies with large samples for the diagnosis and treatment of severe pneumonia during pregnancy have been published. Herein, we report a series of twelve gravid patients with severe pneumonia who were treated at a single institution. Both the clinical features and the maternal-fetal outcomes were reviewed.

## Methods

The retrospective study was conducted at Peking Union Medical College Hospital (PUMCH), which is a tertiary comprehensive hospital and referral center offering medical care for high-risk pregnancies in Beijing, China. We searched the medical records of PUMCH to identify patients who were diagnosed with pneumonia complicating pregnancy between January 2010 and June 2017. We found 51 such patients. The patients’ clinical data including symptoms at presentation, laboratory tests, and treatment strategies were reviewed carefully to screen for severe pneumonia. Severe pneumonia was defined according to the American Thoracic Society guidelines for the management of adults with community-acquired pneumonia as the presence of either one of two major criteria or two of three minor criteria. The major criteria include the need for mechanical ventilation and septic shock. The minor criteria include systolic blood pressure ≤ 90 mmHg, multilobar infiltrates, and PaO_2_/FIO_2_ ratio < 250 [7].

According to this diagnostic standard, we excluded 39 patients who had mild symptoms or who recovered soon after preliminary treatment. Ultimately, 12 gravid patients who were diagnosed with severe pneumonia were included. Their detailed data including demographic data, gestational age at diagnosis, symptoms, physical examinations, laboratory tests, chest radiograph findings, treatment strategies, and obstetric and neonatal outcomes were collected from the medical records. Major medical complications were simultaneously documented. After the data were collected, they were organized into summary forms to indicate the points in common in patients with severe pneumonia.

## Results

### Maternal characteristics and diagnostic evaluations

A total of 12 pregnant women were diagnosed with severe pneumonia at PUMCH during our study period (Table 1). The maternal age ranged between 18 and 36 years, with a mean age of 27 years. Only patient 12 attended regular antenatal care visits at our hospital. The other 11 women were all transferred from other hospitals. Eight women were in their third trimester (29–38 weeks), and the other 4 patients were in their late second trimester (18–25 weeks) at presentation. The mean gestational age was 30 weeks. Anemia was found in six patients (50%) at presentation. The hemoglobin levels of anemic patients ranged from 81 g/L to 93 g/L. Patient 1 was previously diagnosed with membranous nephropathy but was cured 2 years prior to becoming pregnant. Patient 3 had a history of tuberculosis. Patient 7 had a history of severe preeclampsia during her last pregnancy. Patient 8 had chronic hypertension. The remaining eight patients had unremarkable past medical histories.

**Table 1:** Maternal characteristics and clinical presentations

Case	1	2	3	4	5	6	7	8	9	10	11	12	Proportion (%)
Maternal characteristics													
Age (yrs)	26	36	30	24	24	24	31	18	32	32	22	31	NA
Gravidity	1	5	2	1	1	1	2	1	2	3	1	2	NA
Parity	0	1	1	0	0	0	1	0	1	2	0	0	NA
GA (weeks)	18	21^+ 4^	22^+ 5^	25	29	31^+ 3^	32^+ 3^	34	34^+ 4^	36	36^+ 4^	38^+ 6^	NA
Anemia	–	–	+	+	+	+	–	–	–	+	+	–	6/12(50)
Symptoms													
Fever	+	+	+	+	+	+	+	+	+	+	+	+	12/12(100)
Dyspnea	+	+	+	+	+	+	+	+	+	+	+	+	12/12(100)
Cough	–	–	+	+	+	+	+	+	–	+	–	+	8/12(67)
Malaise	–	–	+	+	–	–	–	–	–	–	+	–	3/12(25)
Headache	–	+	–	–	–	–	–	–	–	–	+	–	2/12(17)
Investigations													
CXR/CT evidence	+	+	+	+	+	+	+	+	+	+	+	+	12/12(100)
Leukocytosis	+	–	–	+	–	+	+	+	+	–	+	–	7/12(58)
PaO_2_/FIO_2_	212	200	191	141	207	190	148	73	156	209	133	220	NA
Fever to diagnosis (d)	8	7	68	7	23	5	4	7	10	4	6	4	NA
Pathogen	–	H_7_N_9_	TB	–	TB	–	–	–	–	–	H_1_N_1_	RSV	NA

*NA* not applicable, *GA* gestational age, *CXR* chest X-ray, *CT* computed tomography, *TB* tuberculosis, *RSV* respiratory syncytial virus

All patients had high fevers (> 38 °C) and dyspnea. Eight patients (8/12, 67%) reported a productive cough. Other symptoms, such as chest pain, malaise, and headache, were also reported. Physical examination revealed decreased or bronchial breath sounds in 10 (83%) patients and rales in 10 (83%) patients. Dull percussion was found in patient 2.

Leukocytosis (> 10.0 × 10^9^/L) was observed in 7 patients (58%) at presentation, and leukopenia (< 4.0 × 10^9^/L) was observed in patient 12. Arterial blood gas analysis showed severe hypoxemia in all patients, with PaO_2_/FiO_2_ ratio ranging from 73 to 220. The two patients with the lowest PaO_2_/FiO_2_ ratio both died. Routine screening tests for the causative pneumonia pathogen, such as sputum culture, blood culture, nasal and throat swabs, and serologic testing, were carried out in 11 patients (except for patient 8). Tuberculosis was diagnosed in two patients, and viral pneumonia was diagnosed in three patients; the pathogenic virus was confirmed to be H1N1, H7N9, and respiratory syncytial virus. Patient 8 died within 12 h after admission; thus, none of these tests were carried out. A chest radiograph revealed pulmonary infiltration or consolidation of the bilateral lungs with or without pleural effusion in all 12 patients. For most patients, the disease progression was quick. The time interval between the onset of fever and the confirmation of the diagnosis was typically less than 10 days. For patients 3 and 5, the interval was longer, at 68 and 23 days, respectively. Interestingly, the final diagnoses of these two patients were both tuberculosis.

### Treatment

All patients were admitted to the intensive care unit (ICU) because of severe hypoxemia (Table 2). The length of stay in the ICU ranged from 1 day to 50 days. Mechanical ventilation was performed in all patients, and the duration of mechanical ventilation ranged from 1 day to 48 days. Two patients also received extracorporeal membrane oxygenation (ECMO) therapy for 18 and 12 days. All patients were administered broad-spectrum antibiotics, such as beta-lactams, macrolides, or fluoroquinolones. Antiviral medications, namely, oseltamivir, ganciclovir or acyclovir, were administered to 7 patients (58%). Standard anti-tuberculosis therapy was initiated in patient 3 and patient 5 as soon as tuberculosis was diagnosed. Intravenous courses of pulsed methylprednisolone were administered to 8 patients (67%). Four patients also received intravenous immunoglobulin therapy.

**Table 2:** Treatment received

Case	1	2	3	4	5	6	7	8	9	10	11	12	Proportion (%)
ICU stay (d)	7	50	11	21	6	3	5	1	10	8	21	10	NA
Mechanical ventilation													
Duration (d)	4	48	7	19	6	1	4	1	9	5	21	8	NA
ECMO (d)	–	18	–	–	–	–	–	–	–	–	12	–	NA
Medication													
Antibiotics	+	+	+	+	+	+	+	+	+	+	+	+	12/12(100)
Antivirals	+	+	+	–	–	–	+	–	–	+	+	+	7/12(58)
Anti-TB	–	–	+	–	+	–	–	–	–	–	–	+	2/12(17)
GC	+	+	+	+	+	–	–	–	+	+	–	+	8/12(67)
IVIG	–	+	–	–	–	–	–	–	–	+	+	+	4/12(33)

*ICU* intensive care unit, *ECMO* extracorporeal membrane oxygenation, *TB* tuberculosis, *GC*, glucocorticoids, *IVIG* intravenous immunoglobin

### Complications and outcomes

Two of these patients (patient 8 and patient 11) died of progressive respiratory failure and heart failure (Table 3). The remaining ten patients recovered well. The major complications observed among these patients included septic shock (5/12, 42%), adult respiratory distress syndrome (5/12, 42%), liver failure (4/12, 33%), acute renal failure (4/12, 33%), severe preeclampsia (3/12, 25%), and stress ulcers (2/12, 17%). Patient 4 was diagnosed with the syndrome of inappropriate secretion of antidiuretic hormone (SIADH), and patient 10 was diagnosed with thrombotic thrombocytopenic purpura (TTP).

**Table 3:** Maternal and fetal/neonatal outcomes

Case	1	2	3	4	5	6	7	8	9	10	11	12	Proportion (%)
Maternal outcomes													
Death	–	–	–	–	–	–	–	+	–	–	+	–	2/12(17)
Septic shock	–	+	–	–	+	–	–	+	+	–	+	–	5/12(42)
ARDS	–	+	–	+	–	–	–	–	–	+	+	+	5/12(42)
Liver failure	–	+	–	–	–	–	–	+	–	+	–	+	4/12(33)
Renal failure	–	+	–	–	–	–	–	+	–	+	–	+	4/12(33)
Severe PE	–	–	–	–	–	–	+	+	–	–	+	–	3/12(25)
Heart failure	–	–	–	–	–	–	–	+	–	–	+	–	2/12(17)
Fetal/neonatal outcomes													
IUD	–	+	–	–	–	–	–	+	–	–	–	–	2/12(17)
Preterm birth	NA	NA	NA	NA	+	+	+	NA	+	+	+	–	6/7(86)
CS	NA	NA	NA	NA	–	–	+	NA	+	+	+	+	5/7(71)
Asphyxia	NA	NA	NA	NA	+	–	–	NA	–	–	+	–	2/7(29)
Neonatal death	NA	NA	NA	NA	+	–	–	NA	–	–	–	–	1/7(14)

*ARDS* adult respiratory distress syndrome, *PE* preeclampsia, *IUD* intrauterine death, *CS* cesarean section

Of the 4 patients who presented during their second trimester, 3 patients (patients 1, 3, and 4) chose to terminate their pregnancies after they had recovered from pneumonia for social reasons. Patient 2 experienced intrauterine fetal death and spontaneous miscarriage 40 days after the diagnosis of H7N9 pneumonia. After the miscarriage, the progression of multiple organ dysfunction syndrome (MODS) ceased, and she recovered well. Of the 8 patients who presented during their third trimester, patient 5 had spontaneous preterm labor on the first day of admission, and she delivered a baby with severe asphyxia (Apgar score of 2 at 1 min) who died after several hours. Patient 6 had preterm delivery 30 days after recovery. Patient 8 experienced intrauterine death at presentation. The other 5 patients, whose gestational ages ranged from 31 weeks to 38 weeks, underwent emergency Cesarean section as soon as the diagnosis of severe pneumonia was made. After delivery, the ventilator settings were downregulated for 4 patients, but for patient 11, who had H1N1 pneumonia, no improvement was observed. Although ECMO was used, her cardiorespiratory condition worsened, and she died 21 days later. Her newborn infant had mild asphyxia (Apgar score of 7 at 1 min) but recovered well after primary resuscitation.

## Discussion

The risk of pneumonia during pregnancy appears to be lowest during the first trimester [2]. Advanced gestational age has been proven to be an independent maternal risk factor for pneumonia [6]. Other risk factors for pneumonia in pregnancy include anemia, asthma, smoking, and the use of antepartum corticosteroids and tocolytic agents [8–10]. In this case series, all the patients presented during their second or third trimester with a mean gestational age of 30 weeks. Six patients (6/12, 50%) had anemia at presentation. Among these patients, five (5/6, 83%) had hemoglobin levels below 90 g/L. These findings highlighted the possibility of anemia and advanced gestational age as risk factors for severe pneumonia.

Twenty-five percent of our patients (3/12) had severe preeclampsia, which is much higher than reported prevalence of severe preeclampsia of 0.49% in China [11]. Romanyuk et al. and Chen et al. also reported that women with pneumonia had a higher prevalence of preeclampsia/eclampsia than women without pneumonia [3, 12]. This increased incidence of preeclampsia/eclampsia might be the result of the pathophysiological changes associated with pneumonia. Severe pneumonia is characterized by hypoxemia, which subsequently causes placental hypoxia. The hypoxic placenta releases antiangiogenic and proinflammatory factors that converge upon the maternal endothelium, inducing endothelial dysfunction, hypertension, and organ damage [13]. The three patients complicated with preeclampsia had significantly lower PaO_2_/FIO_2_ ratios than those without preeclampsia (mean value: 118 vs 191, *p* = 0.004), and two of these patients died. Preeclampsia may cause pulmonary edema. Of all obstetric causes of respiratory failure before delivery, severe preeclampsia is the leading cause [14]. A possible explanation for this outcome is that the pulmonary edema caused by severe preeclampsia aggravates the oxygen desaturation caused by pneumonia, predisposing the patient to require a mechanical ventilator. Preeclampsia complicated with pneumonia may be an unfavorable predictive factor for a poor maternal outcome.

Initial misdiagnosis in pregnant patients is not uncommon. When reviewing the clinical data of these 12 patients, we observed that the initial clinical symptoms were similar to those in nonpregnant women, such as fever, cough, malaise, and dyspnea. It is not easy to distinguish between symptoms related to physiological changes and more sinister symptoms of pneumonia during pregnancy. Patient 12 in this case series attended regular antenatal care visits at our hospital; this patient’s pneumonia began with the symptoms of a common cold. Three days later, when she developed dyspnea, labor started. Her respiratory symptoms were intermixed with the discomfort of labor pain. This made the early recognition of pneumonia more difficult. It must be emphasized that close observation is very important; chest radiography should never be withheld from a pregnant woman in whom pneumonia is suspected since the radiation exposure through even multiple diagnostic X-ray procedures or chest CT rarely reaches the dose associated with fetal harm [15].

There are limited publications regarding the timing of delivery. Previous reviews and articles have noted that early delivery is beneficial and potentially life-saving for both mother and fetus [16, 17]. However, earlier on in gestation, the size of the uterus should not affect mechanical ventilation significantly, and the fetus may be nonviable at this younger gestational age. Additionally, the risk of a surgical procedure for a patient in such a critical condition is extremely high. In our case series, pregnancies were continued in 4 patients during 18 to 25 weeks of gestation; the 4 mothers all recovered from pneumonia, but one of them had a spontaneous miscarriage 40 days after the onset of illness. The other 3 pregnancies were uneventful, and live fetuses were discharged from the hospital. Chang et al. and Liao et al. also reported cases of atypical pneumonia requiring mechanical ventilation during the second trimester without complications to either the mother or the newborn [18, 19]. However, during the third trimester, since elective delivery causes a 28% reduction in oxygen requirements within 24 h [17], and since there is a high rate of adverse pregnancy outcomes in maternal pneumonia [3, 20], most case series in the literature suggest an expeditious delivery after 28 weeks of gestation [1, 6]. In our case series, 86% of our patients (6/7) who presented after 28 weeks of gestation with live fetuses underwent delivery soon after the diagnosis of severe pneumonia was made. Their fetal and maternal outcomes were mostly good, except for one neonatal death (patient 5) and one maternal death (patient 11). According to the reported cases in the literature and the experiences of our hospital, it seems reasonable to terminate pregnancies for patients in their third trimester and to continue pregnancies for those in their first or second trimester.

Our study included only 12 patients, and it was a retrospective study. These limitations should be taken into account when interpreting the results of the study.

## Conclusions

In conclusion, severe pneumonia complicating pregnancy was associated with high maternal morbidity and mortality. High incidences of adverse fetal outcomes, such as intrauterine death, preterm birth and cesarean section, were also observed. Anemia, advanced gestational age, and preeclampsia were associated with the severity of pneumonia. Patients with dyspnea, fever and chest pain should be evaluated carefully; chest radiographs should be taken when the diagnosis of pneumonia is highly suspected. Termination of pregnancy is recommended when respiratory function deteriorates progressively despite treatment for patients in their third trimester; it might be reasonable to continue pregnancies for those in their first or second trimester.

## Abbreviations

ARDS: Adult respiratory distress syndrome
CS: Cesarean section
CT: Computed tomography
CXR: Chest X-ray
ECMO: Extracorporeal membrane oxygenation
GA: Gestational age
GC: Glucocorticoids
ICU: Intensive care unit
IUD: Intrauterine death
IVIG: Intravenous immunoglobin
MODS: Multiple organ dysfunction syndrome
NA: Not applicable
PE: Preeclampsia
PUMCH: Peking Union Medical College Hospital
RSV: Respiratory syncytial virus
SIADH: Syndrome of inappropriate secretion of antidiuretic hormone
TB: Tuberculosis
TTP: Thrombotic thrombocytopenic purpura
